# Genetic Predisposition to Colorectal Cancer: How Many and Which Genes to Test?

**DOI:** 10.3390/ijms24032137

**Published:** 2023-01-21

**Authors:** Francesca Rebuzzi, Paola Ulivi, Gianluca Tedaldi

**Affiliations:** Biosciences Laboratory, IRCCS Istituto Romagnolo per lo Studio dei Tumori (IRST) “Dino Amadori”, 47014 Meldola, Italy; francesca.rebuzzi@irst.emr.it (F.R.); gianluca.tedaldi@irst.emr.it (G.T.)

**Keywords:** hereditary colorectal cancer, gene panels, cancer predisposition, Next-Generation Sequencing, cancer risk

## Abstract

Colorectal cancer is one of the most common tumors, and genetic predisposition is one of the key risk factors in the development of this malignancy. Lynch syndrome and familial adenomatous polyposis are the best-known genetic diseases associated with hereditary colorectal cancer. However, some other genetic disorders confer an increased risk of colorectal cancer, such as Li–Fraumeni syndrome (*TP53* gene), *MUTYH*-associated polyposis (*MUTYH* gene), Peutz–Jeghers syndrome (*STK11* gene), Cowden syndrome (*PTEN* gene), and juvenile polyposis syndrome (*BMPR1A* and *SMAD4* genes). Moreover, the recent advances in molecular techniques, in particular Next-Generation Sequencing, have led to the identification of many new genes involved in the predisposition to colorectal cancers, such as *RPS20*, *POLE*, *POLD1*, *AXIN2*, *NTHL1*, *MSH3*, *RNF43* and *GREM1*. In this review, we summarized the past and more recent findings in the field of cancer predisposition genes, with insights into the role of the encoded proteins and into the associated genetic disorders. Furthermore, we discussed the possible clinical utility of genetic testing in terms of prevention protocols and therapeutic approaches.

## 1. Introduction

Colorectal cancer (CRC) ranks third in terms of incidence, but it represents the second cause of cancer-related mortality worldwide [1]. As for other cancers, CRC is the result of a combination of both environmental and genetic risk factors. About one-third of CRCs show familial clustering, but only 5–16% of cases are associated with a germline pathogenic/likely-pathogenic variant in a CRC-predisposition gene [2,3,4,5,6]. Hereditary colorectal cancer (HCRC) includes a group of syndromes that can be divided into two major categories with different clinical features: hereditary non-polyposis colorectal cancer (HNPCC) and hereditary polyposis colorectal cancer (HPCC) [7,8,9]. The incidence of HNPCC conditions is around 3–5% of all CRCs, and the most frequent of these is known as Lynch syndrome, whereas HPCC syndromes account for less than 1% of all CRCs and include adenomatous, hamartomatous, serrated and mixed polyposis syndromes, depending on the histology of the polyps [3,10,11]. Patients carrying pathogenic/likely-pathogenic variants in predisposing genes for CRC are subjected to surveillance according to the National Comprehensive Cancer Network (NCCN) guidelines for HCRC [12]. In the last few years, the advent of Next-Generation Sequencing (NGS) has enabled the analysis of a great number of genes with the advantage of lower costs and wider access to molecular tests for patients with suspected genetic syndromes [13]. In this complex scenario, one of the main issues is to define how many and which genes should be tested in patients with a suspected genetic predisposition to cancer.

In this review, we made a comprehensive description of the genetic syndromes linked to HCRC so far, providing an overview of the main genes involved and the most recent candidate genes for CRC predisposition, including insights into the function of the genes and the recommendations for the management of variant carriers.

## 2. Non-Polyposis Syndromes

### 2.1. Lynch Syndrome

Lynch syndrome (LS) is the most frequent form of HCRC and affects about 3–5% of all CRC diagnoses [4,5,14,15,16,17,18]. It is characterized by an autosomal dominant inheritance pattern and a high risk of developing colorectal (8.7-61%) and extracolonic malignancies (endometrial 30–51%, ovarian 4–15%, gastric 18%, small bowel 3–5%, urinary tract 2–20%, pancreatic 4%, brain or cutaneous cancers) [3,19,20,21,22,23,24,25,26,27]. Phenotypically, CRCs in LS show a small number of adenomatous polyps [3]. LS is caused by pathogenic/likely-pathogenic variants in one of the DNA mismatch repair (MMR) genes, *MLH1*, *MSH2*, *MSH6* and *PMS2* [28], by 3′ deletion of the *EPCAM* gene, which causes the epigenetic silencing of *MSH2*, or by the methylation of the *MLH1* promoter [3,14,15,16,29,30]. MMR proteins coordinately work in sequential steps to initiate the repair of DNA mismatches, constituted by erroneous insertions, deletions and substitutions of bases that can arise during DNA replication and recombination [31]. The germline inactivation of one allele of an MMR gene confers on the individual a high probability of acquiring a second somatic mutation in the wild-type (WT) copy of the corresponding MMR gene during the lifetime. This condition leads to an abnormal DNA mismatch repair function that causes the accumulation of errors during DNA replication, in particular in short repetitive sequences known as microsatellites [5,32]. As a result, the two hallmarks of LS that may help to identify patients potentially affected by this syndrome are microsatellite instability (MSI) and/or loss of MMR protein expression [14,15,30,32]. *MSH2* and *MLH1* represent the most frequently mutated genes in LS [30]. LS patients also have an increased risk of developing several types of tumors. This risk varies by gene, in fact, the carriers of pathogenic variants in *MLH1* and *MSH2* genes have a higher cancer risk and a younger age at diagnosis compared with LS patients with pathogenic variants in *MSH6* and *PMS2* genes [3,20,23]. CRC in LS patients is typified by an accelerated adenoma-carcinoma sequence compared with the natural history of sporadic CRC, probably due to the secondary involvement of mutated tumor suppressor genes and oncogenes because of MMR deficiency (dMMR). Sporadic CRC usually takes about 10 years to develop, while an LS-related CRC takes about two years [3,15,30,33,34].

Guidelines for diagnosing Lynch syndrome have undergone several changes over the years. Initially, the identification of subjects at risk used clinical criteria such as Amsterdam II criteria and revised Bethesda guidelines based on age and family history of cancer [20,35,36]. Due to the limited sensitivity and specificity of the clinical criteria (about 25% of patients truly at risk are lost), the current diagnostic protocol provides a universal tumor screening for LS in which all patients with CRC undergo tumor immunohistochemistry (IHC) of MMR proteins and/or MSI testing. All dMMR cases, excluding those with *BRAF*-V600E mutation or *MLH1* promoter methylation (rarely reported in LS-related tumors), should be tested for germline mutations in MMR genes to confirm their LS diagnosis [14,15,20,32,37,38]. This test is very important because, in addition to the identification of patients with LS, it is able to distinguish patients who may benefit from immunotherapy. Recently, multiple professional organizations (e.g., NCCN) have recommended universal testing for all newly diagnosed LS-associated cancers [3,14,15,38,39,40,41,42].

Regarding surveillance for LS, prevention and early detection of LS-related cancers can increase the survival of these patients. The surveillance protocol could be personalized according to genetic alteration and family history [20]. For LS-related CRC, periodic colonoscopy surveillance is the main technique that allows the resection of polyps and identification of early-stage CRC. For *MLH1*, *MSH2* or *EPCAM* mutation carriers, colonoscopy surveillance is recommended every 1 to 2 years beginning at the age of 20–25 years. Whereas, for *MSH6* and *PMS2* mutation carriers, it should begin at the age of 30–35 years [3,20,24,27]. It is also important to consider the age of cancer onset of the youngest family member, as surveillance should be started approximately 5 years earlier.

Annual transvaginal ultrasound (US), serum CA-125 testing and endometrial biopsy may be considered from age 30 to 35 years for gynecological surveillance. In accordance with the NCCN guidelines, bilateral risk-reducing salpingo-oophorectomy (RRSO) is an option that may be considered and individualized based on whether childbearing is complete, menopausal status, comorbidities, family history, and LS gene, as risks for ovarian cancer vary by mutated gene (there is insufficient evidence to recommend RRSO in *MSH6* and *PMS2* pathogenic variant carriers) [12]. Given the uncertain risk of breast cancer in LS patients, breast surveillance is not recommended but should be evaluated based on family history [12].

Other screening recommendations should also be considered, but there is no clear evidence to support tight surveillance, e.g., upper endoscopy for the risk of LS-related gastric cancer may be considered every 1 to 3 years starting at age 30 for families with gastric cancer aggregation. For pancreatic cancer, an annual magnetic resonance imaging (MRI) and/or endoscopic ultrasound (EUS) should be considered in LS patients with at least one first-degree relative with pancreatic cancer [3,20,43].

Finally, patients with LS that have an impairment of the MMR system can be treated with anti-PD-1/PD-L1 therapy [44,45,46] and also can benefit from chemoprevention based on the daily use of 600 mg of aspirin for at least 2 years. This therapy has shown a 60% reduction in the incidence of CRC and other LS-related cancers, as demonstrated by the Colorectal Adenoma/Carcinoma Prevention Programme 2 (CAPP2) [3,20,47,48,49].

### 2.2. Turcot Syndrome

Turcot syndrome (TS) represents the combination of primary brain tumors and CRC in a patient with either LS or FAP. TS can be caused by biallelic mutations in the MMR genes (Turcot syndrome 1, TS1) or by monoallelic mutations in the *APC* gene (Turcot syndrome 2, TS2). Regarding the origin of brain tumors in TS, glioblastomas are associated with MMR gene mutations, specifically in the *MLH1* gene, whereas medulloblastomas are associated with *APC* gene mutations (about 40% of patients with TS develop a medulloblastoma).

Phenotypically, TS1 has fewer polyps in the colon (less than 100) and can also present with hematologic malignancies, café au lait spots and glioblastoma multiforme. On the opposite, TS2 results in very significant polyposis of the colon, with polyps numbering in the thousands, and also presents epidermal cysts, tumors in other areas of the body and medulloblastoma [11]. There is a lack of guidelines in the surveillance of patients with TS, for which family history is of particular importance. Serial colonoscopies should be performed in patients with a family history of FAP or LS. Furthermore, it is recommended that upper gastrointestinal surveillance is performed every 3 years for cancers in the duodenum of patients undergoing colectomy. Screening for brain tumors is difficult because imaging once a year does not seem to be enough, given the aggressiveness of medulloblastomas. For this reason, special attention should be made to neurologic signs. However, screening guidelines for FAP and LS are followed, considering the association of TS with these pathologies [50,51,52,53].

### 2.3. Muir–Torre Syndrome

Muir–Torre syndrome (MTS) is an autosomal dominant variant of LS, that is observed in 9.2% of patients with HNPCC. In addition, MTS is caused by pathogenic variants in MMR genes but differs from LS in some clinical manifestations. The genes most commonly mutated in MTS include *MLH1*, *MSH2*, *MSH6* and *PMS2*. MTS is characterized by sebaceous neoplasms of the skin and visceral malignancies, with colonic carcinoma being the most common. Other cancers have been described in association with MTS, including malignancies involving the endometrium, cervix, ovaries, breast, small bowel, bone, hepatobiliary tract, brain, pancreas, upper uroepithelial tract, blood (lymphoma and leukemia) and lung. More recently, some autosomal recessive cases of MTS have been described, which do not display MSI and are due to defects in the *MUTYH* gene. Regarding surveillance in MTS patients, once the diagnosis has been made, patients should undergo annual surveillance for visceral and cutaneous malignancy and upper and lower gastrointestinal endoscopy. Colonoscopy may begin as early as 18 years of age, while upper endoscopy is recommended around 25–30 years [8,54,55,56,57,58].

### 2.4. Constitutional Mismatch Repair Deficiency Syndrome

Constitutional mismatch repair deficiency syndrome (CMMRD) is an autosomal recessive disease characterized by homozygous or compound heterozygous germline pathogenic variants in MMR genes (*MLH1*, *MSH2*, *MSH6* and *PMS2*). This genetic condition is associated with a high risk of developing CRC, small bowel, hematological, brain, endometrium and urinary tract tumors at an early age (mean age at diagnosis: 16 years). Criteria for the diagnosis of CMMRD were defined by the European Care for CMMRD Consortium. The current guidelines recommend colonoscopy surveillance starting at the age of 8 years, with a semestral blood test, abdominal US and annual brain MRI [15,20,59,60,61,62].

### 2.5. Lynch-Like Syndrome

The main feature of LS is MMR deficiency (dMMR), but 10–15% of CRCs with dMMR are not associated with LS. This syndrome is distinguished from classic LS because it presents dMMR but lacks a germline mutation. In fact, this molecular dysfunction could be caused by (I) somatic methylation of *MLH1* promoter or (II) double somatic pathogenic variants in the MMR genes. The latter cause accounts for 50–70% of this population and is identified as Lynch-like syndrome. Given the sporadic nature of this pathological condition, patients with Lynch-like syndrome are characterized by cancers at more advanced age than LS patients and present a fewer frequency of family history of LS-related tumors. However, intensive surveillance of LS-associated tumors in patients and relatives potentially at risk is strongly recommended [15,20,30,63,64].

### 2.6. Familial Colorectal Cancer Type X Syndrome

Familial colorectal cancer type X (FCCTX) is an autosomal dominant condition that accounts for up to 40% of families who fulfill Amsterdam criteria for HNPCC but do not present MSI tumors or germline MMR gene alterations [65]. Despite the identification of multiple candidate genes, only pathogenic variants in the *RSP20* gene (chromosome 8q12.1), encoding a ribosomal protein that is a component of the 40S subunit [66], have been unequivocally linked to the disease. Only recently have researchers identified the involvement of this gene in HNPCC [67], thanks to the advent of sequencing-based techniques. However, studies on the role of these mutations in carcinogenesis are still ongoing. This disease causes a significantly increased risk for CRC, albeit lower than LS. Moreover, tumors in FCCTX have a later age of onset than LS. Compared with LS patients, FCCTX has a greater adenoma/carcinoma ratio, suggesting a slower process of carcinogenesis. For this reason, a colonoscopy every 3–5 years, starting 10 years before the age at diagnosis of the youngest affected relatives, may be considered [3,15,20,68,69,70,71,72,73].

### 2.7. Li–Fraumeni Syndrome

The Li–Fraumeni syndrome (LFS) is a rare autosomal dominant disorder characterized by a high predisposition to several types of cancer, such as brain tumors, sarcomas, acute leukemia and adrenocortical tumors [74,75,76,77,78,79,80,81,82].

The syndrome is caused by pathogenic variants in the *TP53* gene (chromosome 17p13.1), encoding the p53 protein, a tumor suppressor that responds to different cellular stresses to regulate expression of target genes, thereby inducing cell cycle arrest, apoptosis, senescence, DNA repair or metabolism changes [83].

The lifetime risk of CRC for patients with LFS is estimated to be higher than 20% [12,54,55,84,85]. Given the risk of developing gastrointestinal cancers, the NCCN guidelines suggest that carriers of *TP53* pathogenic variants should undergo upper endoscopy and colonoscopy every 2–5 years starting from age 25 years [39]. Moreover, in children, the recommendations are to perform a clinical examination and abdominal ultrasound every 6 months and an annual whole-body MRI and brain MRI from the first year of life if the *TP53* variant is known to be associated with childhood cancers. In adults, the surveillance should include a clinical examination every year, whole-body MRI, breast MRI in females from 20 to 65 years and brain MRI to 50 years [56].

## 3. Adenomatous Polyposis Syndromes

This class of polyposis syndromes presents adenomatous polyps. A polyp is an elevation of the mucosal surface that can occur anywhere in the gastrointestinal tract [57]. Adenomatous polyps, also known as adenomas, originate in epithelial tissues (the thin layer covering organs, cavities, and vessels of the body). There are three histologic types of adenomatous polyps: tubular (containing >75% glandular elements), villous (>75% villous elements) and tubulovillous (>25% of both glandular and villous elements) [58,86].

### 3.1. Familial Adenomatous Polyposis and Attenuated Familial Adenomatous Polyposis

Pathogenic or likely-pathogenic germline alterations in the *APC* gene (chromosome 5q22.2) are associated with familial adenomatous polyposis (FAP) [87,88]. The APC protein is a tumor suppressor that acts as a Wnt signaling antagonist and regulates transcriptional activation, cell migration and apoptosis [89]. FAP is characterized by polyposis and carcinomas of the colon, as well as extra-colonic carcinomas, such as duodenal and gastric cancers, desmoid tumors, osteomas, hepatoblastoma, epidermoid cysts and papillary thyroid carcinoma [40,88]. This autosomal dominant syndrome represents <1% of all cases of CRC and constitutes the most frequent cause of polyposis. In approximately 30–40% of FAP cases, *APC* mutation has a de novo origin, whereas 20% of FAP patients present somatic mosaicism [15,20,90,91]. The severity of the disease is associated with the mutation site in the *APC* gene: mutations located between codons 1250 and 1464 have been associated with more severe forms of FAP, whereas mutations at the end of the gene or in exon nine are associated with a mild polyposis phenotype called attenuated familial adenomatous polyposis (AFAP) [15,30,92]. In fact, classical FAP predisposes to hundreds to thousands of colonic and rectal polyps that may develop into colorectal carcinoma with a 100% risk before age 50 without any intervention. Conversely, the attenuated form is characterized by fewer polyps (10–100 adenomas) and later onset of both polyps and carcinomas, as well as a decreased CRC risk (70–80% by age 80 if untreated) [3,15,40,90,93]. 

FAP and AFAP also predispose to other cancers with a lifetime risk ranking in a range between: 0.1–7.1% for gastric cancer, 1–10% for duodenal and small bowel cancers, 10–24% for desmoid tumors, 1.2–12% for thyroid cancers, 0.4–2.5% for hepatoblastoma and 1–2% for pancreatic cancer [3,12,94].

To confirm the diagnosis of FAP or AFAP, germline testing to evaluate for a pathogenic variant in the *APC* gene is recommended. Germline testing is important to differentiate between other types of adenomatous polyposis for the consideration of screening, counseling, risk assessment and testing of family members. Surveillance is similar for classical and attenuated forms and it should be performed in all mutation carriers as well as in all family members considered at risk. For FAP patients, an annual colonoscopy beginning at age 10–15 is recommended, while for AFAP patients, a later starting age can be considered (18–20 years). Gastroduodenal endoscopy should begin at age 20–25 with a period of follow-up varying from every 3 months to 5 years, depending on the burden of polyps. Furthermore, guidelines recommend a thyroid ultrasound starting from the late teenage years and repeating every 2–5 years. If a family history of desmoid tumors is present, the guidelines suggest abdominal computed tomography (CT) with contrast or MRI annually. Finally, hepatoblastoma surveillance is proposed by liver palpation, abdominal ultrasound and serum alpha-fetoprotein every 3–6 months during the first 5 years of life. These patients usually undergo surgery by the age of 25 years, which represents the cornerstone of FAP/AFAP management. Surgical treatment includes total proctocolectomy (TPC) with end ileostomy or with restoration via an ileal pouch-anal anastomosis (IPAA) or total abdominal colectomy (TAC). Close monitoring needs to continue post-colectomy, with annual endoscopic evaluation [3,12,20,95,96,97].

Chemoprevention in FAP/AFAP patients with the use of non-steroidal anti-inflammatory drugs (NSAIDs) has been shown to reduce the number and extent of colorectal adenomas. Given the cardiovascular risk correlated with the use of these drugs, NSAIDs have not yet been approved [12,20,98,99,100].

In 2012, gastric adenocarcinoma and proximal polyposis of the stomach (GAPPS) were described as a new autosomal dominant syndrome [101]. The key clinical features of GAPPS include fundic gland polyposis (FGP) of the stomach with occasional hyperplastic and adenomatous polyps, focal foveolar-type dysplasia, hyperproliferative aberrant pits and development of adenomas with gastric-type dysplasia or intestinal-/mixed-type gastric adenocarcinoma [101,102,103]. In 2016, point mutations in *APC* promoter 1B that co-segregated with the disease in all three families were identified [104]. Therefore, GAPPS is now considered a part of the broad phenotypic spectrum of *APC*-associated polyposis syndromes. In GAPPS, the polyps are mainly located in the stomach; However, some patients showed the presence of colorectal polyps, and consequently, testing for mutations in the *APC* promoter 1B should be taken into account in patients with gastrointestinal polyposis negative for the presence of genetic alterations in the coding regions of *APC* gene.

### 3.2. MUTYH-Associated Polyposis

The *MUTYH*-associated polyposis (MAP) is characterized by a recessive inheritance pattern with a wide range of phenotypes, including adenomatous polyposis (<100 polyps), seldom including cases with no polyps at the time of CRC diagnosis [15,105,106]. In comparison with FAP/AFAP, MAP presents a lower risk of extracolonic manifestations (up to 38% lifetime risk, primarily small bowel) and duodenal adenomas [3,107].

This syndrome is caused by biallelic pathogenic variants in the *MUTYH* gene (chromosome 1p34.1), encoding the MutY DNA glycosylase, involved in oxidative DNA damage repair and, if unrepaired, apoptosis signaling [108]. As a result, MAP-associated tumors are MMR proficient (MSI may also be detected due to somatic alterations of MMR genes, explaining some Lynch-like cases) and are enriched of G:C to T:A transversions [15,109].

The risk of developing CRC ranges from 70% to 90% for *MUTYH* biallelic variant carriers. Individuals who have a monoallelic *MUTYH* mutation are at slightly increased CRC risk (1.5–2 fold to the general population) but do not show a polyposis phenotype [3,12,15].

Current surveillance measurements for CRC risk in *MUTYH* biallelic variant carriers include a high-quality colonoscopy every 1–2 years, beginning no later than age 25–30 years [110]. If the adenoma burden cannot be endoscopically handled, then a colectomy with ileorectal anastomosis (IRA) is recommended. A proctocolectomy with IPAA should be considered if rectal involvement is substantial. If the patient had a colectomy with IRA, then endoscopic evaluation of the rectum should be scheduled every 6–12 months, depending on polyp burden [12]. Chemoprevention may be considered in selected patients, but options have not been studied specifically in MAP [12]. In regard to extracolonic manifestations, an annual physical examination and baseline upper endoscopy (including complete visualization of the ampulla of Vater) beginning at age 30–35 years should be considered [3,12,20].

### 3.3. Polymerase Proofreading-Associated Polyposis

Polymerase proofreading-associated polyposis (PPAP) is a syndrome characterized by a high risk of CRC and an increased risk of breast, duodenal, ovarian and central nervous system tumors [111,112]. Other cancers, including pancreatic cancer, gastric cancer and gastrointestinal stromal tumor (GIST), have been reported in a small number of individuals [111,112,113,114,115].

The syndrome is caused by germline pathogenic variants in the *POLE* gene (chromosome 12q24.33), encoding the catalytic subunit of DNA polymerase epsilon, and in the *POLD1* gene (chromosome 19q13.33), encoding the catalytic subunit of DNA polymerase delta, both involved in proofreading activity of newly synthesized DNA for replication errors [116]. This function is essential for replication fidelity, and its disruption by pathogenic variants leads to the accumulation of thousands of mutations in the tumors, which is called an ultramutated phenotype [15,117,118]. Proofreading deficiency has been associated with a good prognosis and excellent response to immune checkpoint inhibition [119]. The risk for CRC is estimated to be higher than 20% (current estimates range is 21–28% for *POLE* and 80–90% for *POLD1* variant carriers) [12,113]. For this reason, carriers of pathogenic variants in *POLE* and *POLD1* genes should undergo a surveillance protocol, including a high-quality colonoscopy starting at age 25–30 years, to be repeated every 2–3 years if negative. If polyps are found, a high-quality colonoscopy should be performed every 1–2 years with consideration of surgery if the polyp burden becomes unmanageable [12].

### 3.4. AXIN2-Associated Polyposis

The *AXIN2*-associated polyposis is a syndrome characterized by colorectal polyposis and oligodontia (congenital absence of more than six teeth) [120,121,122]. The syndrome presents an autosomal dominant pattern, and it is caused by pathogenic variants in the *AXIN2* gene (chromosome 17q24.1), encoding the Axin-related protein, playing an important role in the regulation of the stability of beta-catenin in the Wnt signaling pathway [123]. The colorectal phenotype is highly variable; affected individuals have been reported with zero to 100 adenomatous polyps. Not all patients have oligodontia, and pathogenic variants in exon seven are associated with this phenotype [121,124,125]. The risk of CRC in *AXIN2* pathogenic variant carriers is not precisely estimated; however, these patients should undergo a surveillance protocol, including a high-quality colonoscopy starting at age 25–30 years, to be repeated every 2–3 years if negative. If polyps are found, the patients should undergo a high-quality colonoscopy every 1–2 years with consideration of surgery if the polyp burden becomes unmanageable by colonoscopy [12].

### 3.5. NTHL1 Tumor Syndrome

*NTHL1* tumor syndrome is a recessive disorder characterized by adenomatous and small bowel polyps (range 1–100) [126,127,128,129,130,131,132]. The syndrome is caused by biallelic pathogenic variants in the *NTHL1* gene (16p13.3), encoding a DNA N-glycosylase of the endonuclease III family. Biallelic *NTHL1* mutations are associated with an increased risk for CRC (>20%) characterized by an excess of C > T transitions. The risk of extracolonic manifestations is between 6–56% by age 60, including breast cancer (most common, can be early onset), endometrial, skin, urothelial, brain, pancreas, ovary, head and neck and hematologic malignancies [132,133,134,135].

Monoallelic pathogenic variants in the *NTHL1* gene do not appear to be associated with an increased risk of polyposis and/or CRC [136]. Guidelines for the medical management of individuals with biallelic *NTHL1* mutations have been developed by the NCCN and recommend a high-quality colonoscopy starting at age 25–30 years to be repeated every 2–3 years if negative, and every 1–2 years if polyps are identified. Furthermore, surgery should be considered if the polyp burden becomes unmanageable [12]. Currently, there are no specific guidelines for female breast cancer risk. However, the possibility of an increased risk warrants consideration of individualized breast cancer risk-reduction strategies, such as the modification of standard population screening recommendations by starting screening at a younger age and/or performing screenings at a greater frequency [39,137]. These guidelines will evolve as we learn more, and it is recommended that patients with biallelic *NTHL1* mutations be managed by a multidisciplinary team with expertise in medical genetics and the care of patients with hereditary cancer syndromes.

### 3.6. MSH3-Associated Polyposis

Biallelic mutations in the *MSH3* gene have been found in a small number of individuals with recessive adenomatous polyposis (>20 polyps) and a history of CRC and other cancers (brain, stomach, thyroid and small bowel) [138]. The *MSH3* gene (chromosome 5q14.1) encodes a protein that forms a heterodimer with MSH2 to form MutS beta, part of the post-replicative DNA mismatch repair system [139].

Although there are no precise estimates of the CRC risk associated with biallelic *MSH3* mutations yet, this risk may be significantly increased over that in the general population [12,138]. Consequently, guidelines suggest performing a high-quality colonoscopy starting at age 25–30 years to be repeated every 2–3 years if negative. If polyps are found, a high-quality colonoscopy every 1–2 years is recommended with consideration of surgery if the polyp burden becomes unmanageable [12].

## 4. Hamartomatous Polyposis Syndromes

This type of syndrome presents characteristic polyps called hamartomatous polyps, composed of the normal cellular elements of the gastrointestinal tract but with a markedly distorted architecture. They vary in size and may have a characteristic histological structure [140,141,142].

### 4.1. Peutz–Jeghers Syndrome

Peutz–Jeghers syndrome (PJS) is an autosomal dominant disorder characterized by melanocytic macules on the lips, buccal mucosa and digits and by multiple gastrointestinal hamartomatous polyps [143]. PJS polyps are typically multilobulated with a papillary surface and branching bands of smooth muscle covered by hyperplastic glandular mucosa, often forming a distinctive tree-like configuration [12,15,144]. The syndrome is caused by pathogenic variants in the *STK11* gene (formerly *LKB1*), located on chromosome 19p13.3 and encoding a serine/threonine kinase that regulates energy metabolism and cell polarity [145,146]. The lifetime risk for CRC corresponds to 39% (Table 1) [12,40,147,148,149]. Moreover, PJS patients have an increased risk for a variety of extracolonic malignancies, such as breast (32%), uterus (9%), ovary (21%), pancreas (11–36%), stomach (29%), small bowel (13%), testis (9%) and lung tumors (7–17%). A clinical diagnosis of PJS can be made when an individual has two or more of the following features: (I) two or more PJS polyps of the small intestine, (II) typical mucocutaneous hyperpigmentation and (III) a family history of PJS [15,150]. For this reason, the NCCN guidelines suggest that PJS patients should undergo upper endoscopy and colonoscopy with polypectomy every 2–3 years, starting at the age of 18. Shorter intervals may be indicated based on polyp size, number and pathology. Small bowel visualization is also recommended every 2–3 years starting at age 18, while pancreatic surveillance by endoscopic ultrasound or MRI should be conducted annually. Finally, women should have mammography and a Pap smear annually, starting at age 30 and 18–20, respectively [12,151].

### 4.2. PTEN Hamartoma Tumor Syndrome

The *PTEN* hamartoma tumor syndrome (PHTS) includes Cowden syndrome, Bannayan–Riley–Ruvalcaba syndrome, *PTEN*-related Proteus syndrome and *PTEN*-related Proteus-like syndrome [156]. These syndromes are associated with pathogenic variants in the *PTEN* gene, located on chromosome 10q23.31 and encoding a phosphatidylinositol-3,4,5-trisphosphate 3-phosphatase that antagonizes the PI3K signaling pathway and negatively regulates the MAPK pathway [160].

Cowden syndrome (CS) is an autosomal dominant disorder characterized by benign hamartomas as well as by an increased lifetime risk of breast, thyroid, uterine, colorectal and other cancers [156,161,162]. Upper or lower gastrointestinal polyps occur in more than 90% of individuals with a *PTEN* pathogenic variant [163]. The lifetime risk of CRC in *PTEN* pathogenic variant carriers is estimated to be 11–20% [142,162,164,165,166,167,168]. Consequently, in accordance with the NCCN guidelines, carriers of *PTEN* pathogenic variants should undergo colonoscopy starting at age 35 years, unless symptomatic or if there is a close relative with CRC before age 40 years, then 5–10 years before the earliest known CRC in the family. If the patient is symptomatic or polyps are found, a colonoscopy should be performed every 5 years or more frequently, depending on the degree of polyposis identified [39].

Recently, also germline pathogenic variants in *PIK3CA* and *AKT1* genes have been reported as associated with CS [169], but the role of these genes in the pathogenesis of the disease is not well established.

### 4.3. Juvenile Polyposis Syndrome

Juvenile polyposis syndrome (JPS) is an autosomal dominant disorder that predisposes to hamartomatous polyps in the gastrointestinal tract, specifically in the stomach, small intestine, colon and rectum [157]. The majority of juvenile polyps are benign; however, they can undergo malignant transformation. The term “juvenile polyps” refers to special histopathology and not the age of onset, as the polyp might be diagnosed at all ages. The juvenile polyp has a spherical appearance and is microscopically characterized by the overgrowth of an edematous lamina propria with inflammatory cells and cystic glands.

JPS is caused by pathogenic variants in the *BMPR1A* gene (chromosome 10q23.2), encoding the bone morphogenetic protein receptor type IA, and in the *SMAD4* gene (chromosome 18q21.2), encoding a member of the Smad family of signal transduction proteins, both involved in TGF-β/BMP signaling pathway [170,171].

Lifetime estimates of developing gastrointestinal cancers in families with JPS range from 11% to 86%, with variability by region, time period included, and associated gene [172,173,174,175,176]. In fact, approximately 15% of JPS individuals develop cancer [175,177]. The risk of colorectal cancer ranges between 17% and 22% by 35 years of age and approaches 68% by 60 years of age [178]. In the JPS context, small bowel and pancreatic cancers have also been reported [179,180,181,182,183]. Individuals with *SMAD4*-related JPS are more likely to have a personal or family history of upper gastrointestinal polyps than individuals with a *BMPR1A* pathogenic variant.

According to the clinical practice guidelines for JPS, individuals with a *BMPR1A* or *SMAD4* pathogenic variant should undergo a high-quality colonoscopy with polypectomy and upper endoscopy with polypectomy, starting at 12–15 years and repeating every 2–3 years if polyps are found. Shorter intervals may be indicated based on polyp size, number and pathology [12,61,184].

## 5. Serrated Polyposis Syndromes

These clinical conditions are characterized by the presence of typical polyps called serrated polyps. Serrated polyps have a particular growth pattern sticking out from the surface of the colon or rectum. The polyps are defined by their saw-toothed appearance under the microscope [185]. These polyps show serrated, dilated crypts with branching at the base, enlarged hyperchromatic nuclei, and normally arranged, small basilar nuclei [186]. There are different types of serrated polyps [187], including:

- Hyperplastic polyps, the most common and are usually located on the left side of the colon and are not usually precancerous;
- Sessile serrated polyps (SSPs), which are bigger and are located on the right side and can turn into cancer somewhat quickly;
- Serrated adenomas are less common but have dysplasia (abnormal cells) and can progress to cancer.

### RNF43-Associated Serrated Polyposis

The *RNF43*-associated serrated polyposis is a genetic disorder characterized by serrated polyposis (5–100 polyps) and an increased risk of CRC, not precisely estimated [188,189,190,191,192]. The syndrome is caused by pathogenic variants in the *RNF43* gene (chromosome 17q22), encoding a RING-type E3 ubiquitin ligase that negatively regulates Wnt signaling [193].

There are no specific surveillance recommendations for *RNF43* pathogenic variant carriers, but the risk of CRC is elevated. Consequently, the recommendations for patients with serrated polyposis syndrome include a high-quality colonoscopy with polypectomy until all polyps ≥5 mm are removed, then a colonoscopy every 1 to 3 years, depending on the number and size of polyps. Clearing all polyps is preferable but not always possible. Surgical referral should be considered if colonoscopic treatment and/or surveillance are inadequate [12]. The risk of CRC in first-degree relatives of individuals with serrated polyposis is elevated. Consequently, they should undergo colonoscopy starting at the age of 40 years or at the same age as the youngest diagnosis of serrated polyposis, if uncomplicated by cancer, or ten years earlier than the earliest diagnosis in a family with CRC secondary to serrated polyposis [12].

## 6. Mixed-Type Polyposis Syndromes

Mixed-type polyposis syndromes are characterized by the presence of multiple colorectal polyps of different histopathological types (adenomatous, hyperplastic and hamartomatous) [194].

### GREM1-Associated Mixed Polyposis

The *GREM1*-associated mixed polyposis syndrome is a genetic disorder characterized by an increased risk of CRC (estimated to be 11–20%) [12,195,196].

The syndrome is caused by pathogenic duplications in the *GREM1* gene (chromosome 15q13.3), encoding a member of the bone morphogenetic protein (BMP) antagonist family, which plays a role in regulating organogenesis, body patterning and tissue differentiation [197].

NCCN guidelines suggest that *GREM1* variant carriers should undergo a high-quality colonoscopy starting at age 25–30 years to be repeated every 2–3 years if negative. If polyps are found, a high-quality colonoscopy every 1–2 years, with consideration of surgery if the polyp burden becomes unmanageable, should be performed [12].

## 7. Other Emerging Genes

Thanks to the wide use of NGS, the number of genes that have been associated with a CRC risk has hugely increased in the last few years. However, the most critical issue is the penetrance estimate of these genetic variants, and further studies are needed to confirm these associations.

Indeed, other genetic alterations have been reported in CRC families, especially in genes involved mainly in breast cancer predisposition.

In particular, *BRCA1* and *BRCA2*, the main genes involved in hereditary breast and ovarian cancer, encoding proteins involved in homologous recombination, have been recurrently associated with a CRC risk that, however, is still debated and not precisely estimated [198,199,200,201,202].

Additionally, pathogenic variants in the *CHEK2* gene have been associated with CRC risk, estimated to be 5–10%, though heterogeneity may exist based on the type of *CHEK2* pathogenic variant [203,204,205,206,207,208]. This gene encodes a nuclear serine/threonine kinase that acts as a tumor suppressor in different cellular processes [209]. For *CHEK2* variant carriers with a personal history of CRC, guidelines suggest following the recommendations for post-CRC resection [12]. For *CHEK2* variant carriers without a personal history of CRC, a high-quality colonoscopy screening every 5 years, beginning at age 40 or 10 years before the earliest CRC diagnosis among first-degree relatives, is recommended [12]. Female patients with a *CHEK2* pathogenic variant also have an increased risk of breast cancer that is estimated to be 20–40%; consequently, they should undergo annual mammography starting at age 40 years and breast MRI with contrast starting at age 30–35 years [39].

Germline pathogenic variants in the *ATM* gene have also been recurrently associated with an increased CRC risk that has been estimated at 5–10% [210,211,212,213]. This gene encodes a phosphatidylinositol 3-kinase protein that acts as a tumor suppressor and responds to DNA damage by phosphorylating key substrates involved in DNA repair and cell cycle control [214]. Biallelic pathogenic variants in the *ATM* gene are associated with ataxia-telangiectasia, an autosomal recessive disorder characterized by cerebellar ataxia, telangiectases, immune defects and a predisposition to malignancy [215]. However, monoallelic pathogenic variants are associated with an increased risk of CRC, breast, ovarian and pancreatic cancer [216,217,218,219,220,221,222,223]. There is currently insufficient evidence to provide specific CRC risk management recommendations for carriers of an *ATM* heterozygous variant, so this should be based on family history [12].

Germline pathogenic variants in the *BLM* gene have been recurrently associated with an increased CRC risk that has been estimated at 5–10% [213,224,225]. This gene encodes a DNA helicase RecQ protein involved in homologous recombination, and its biallelic mutations are associated with Bloom syndrome, a recessive disorder characterized by severe pre- and post-natal growth deficiency, immune abnormalities, sensitivity to sunlight, insulin resistance and high risk for many cancers that occur at an early age [226]. There is currently insufficient evidence to provide specific CRC risk management recommendations for carriers of a *BLM* heterozygous variant, so this should be based on family history [12].

In 2009, germline pathogenic variants in the *GALNT12* gene were associated with an increased CRC risk that has been estimated at 5–10% [227,228,229,230]. This gene encodes an enzyme involved in glycosylation [231]. There is currently insufficient evidence to provide specific CRC risk management recommendations for carriers of a *GALNT12* pathogenic variant, so this should be based on family history [12].

Recently, biallelic variants in the *MLH3* gene, encoding a protein involved in the MMR system [232], have been associated with CRC predisposition [233]. The CRC risk for these patients is not precisely estimated; however, NCCN guidelines recommend similar management strategies as described for carriers of *AXIN2* pathogenic variants [12].

Moreover, in 2018, a systematic review of The Cancer Genome Atlas (TCGA) data was performed focusing on pathogenic germline variants: in addition to *MSH6* and *APC* genes, pathogenic variants in *BRCA1*, *BRCA2*, *ATM*, *PALB2*, *SDHA* and *RET* genes have been detected in colon and rectum adenocarcinoma patients [234]. Some of these genes are intriguing candidates in the predisposition to CRC and, in the future, could be included in diagnostic panels for the assessment of cancer risk.

## 8. Conclusions

CRC is one of the most common and deadly tumors, and among risk factors for the development of this cancer, genetic predisposition plays an important role.

Some syndromes, such as LS and FAP, have been known for decades as diseases predisposing to CRC. Consequently, for carriers of pathogenic variants in the associated genes, accurate cancer risk estimates are available, as well as surveillance protocols for cancer prevention and early detection of the disease.

In addition to LS and FAP, there are some genetic disorders, such as LFS, MAP, PJS, JPS and PHTS (Table 1), that confer a high risk of CRC. Consequently, these genes should be included in a panel of genes for the identification of patients at risk of CRC. Moreover, there are some other genes, such as *RPS20*, *POLE*, *POLD1*, *AXIN2*, *NTHL1*, *MSH3*, *RNF43* and *GREM1*, that are classified as moderate or low penetrance genes for the risk of CRC (Table 1); however, the increasing evidence of associated cancer risks suggest that they should be analyzed, whenever possible, in patients with a suspected CRC predisposition, especially the ones who have tested negative for the other syndromes.

On the whole, it is indisputable that NGS has deepened our knowledge of CRC predisposition by increasing the number of susceptibility genes. However, because of the growing demand for higher throughput and lower costs, standardized procedures need to be carefully assessed. Moreover, genetic counseling and risk evaluation, as well as clinical management of patients and families at risk, are becoming more and more challenging. In particular, all healthcare professionals who offer genetic testing must engage in constant education as the field is continuously evolving, with new data becoming available. An important aspect is the selection of patients for the genetic test, which is currently based on the number and type of cancers in the family and on the age of onset of these tumors. The main cancer syndromes have guidelines to select patients for the genetic test, but with the increase in the number of predisposing genes, they should be improved to identify individuals who can really benefit from a genetic test and, at the same time, to avoid the overuse of genetic tests.

Indeed, the diagnostic use of multigene panels, instead of the traditional single-gene analysis, generates many advantages as well as some critical issues. Before the advent of NGS, turn-around times for genetic testing were long, in some cases more than 6–12 months, while nowadays, new technologies provide results in less than a month in many cases. This short time is extremely useful for the affected individuals because the result of the genetic test can address or modify the surgical and therapeutic approach to the disease, but at the same time, it can generate issues in genetic counseling and the management of the family, since the implications of cancer predisposition need time to be properly understood by the patient and family members.

When taking into account all these considerations, it is clear how difficult it is to find the right combination of genes to be tested in patients with a suspected genetic predisposition to CRC [235]. At the international level, efforts are being made to achieve a consensus [236], but the identification of a balance between costs for health systems and benefits for patients remains one of the biggest challenges for the future. Moreover, larger case-control studies are needed to better refine the penetrance estimates and to evaluate the correct preventive and therapeutic approach for each patient.

In summary, new genes are constantly emerging from NGS studies [237,238,239], showing that CRC predisposition is distributed over many genes, with only a few genes being recurrently mutated. However, testing on genes other than MMR and *APC*/*MUTYH* is not routinely performed due to the lack of information about the actual risks for pathogenic variant carriers and the unavailability of surveillance programs. These findings address the choice of wide panels, including the genes involved in the main cancer syndromes and, possibly, the new emerging genes. This approach creates new diagnostic opportunities [240] and can guide not only the choice of the best chemoprevention and prophylactic surgeries but also the choice of novel targeted therapies and personalized treatments based on the genetic characteristics of each patient.

## Figures and Tables

**Table 1:** List of the main syndromes associated with CRC with associated genes and CRC risk estimates.

HCRC Type	Syndrome	Associated Genes	Locus	Inheritance	CRC Risk *	References
**Hereditary non-polyposis colorectal cancer**						
	Lynch syndrome (LS)	*MLH1*	3p22.2	AD	46–61%	[19]
		*MSH2*	2p21-p16		33–52%	
		*MSH6*	2p16.3		10–44%	
		*PMS2*	7p22.1		8.7–20%	
		*EPCAM*	2p21		33–52%	
	Constitutional mismatch repair deficiency syndrome (CMMRD)	MMR genes	-	AR	NA	[152]
	Familial colorectal cancer type X syndrome (FCCTX)	*RPS20*	8q12.1	AD	NA	[67]
	Li–Fraumeni syndrome (LFS)	*TP53*	17p13.1	AD	>20%	[74]
**Hereditary polyposis colorectal cancer**						
**Adenomatous**	Familial adenomatous polyposis (FAP)	*APC*	5q22.2	AD	100%	[87]
	Attenuated familial adenomatous polyposis (AFAP)	*APC*	5q22.2	AD	70%	[87]
	*MUTYH*-associated polyposis (MAP)	*MUTYH*	1p34.1	AR	70–90%	[105]
	*NTHL1*-associated polyposis	*NTHL1*	16p13.3	AR	>20%	[126]
	*MSH3*-associated polyposis	*MSH3*	5q14.1	AR	NA	[153]
	Polymerase proofreading-associated polyposis (PPAP)	*POLE*	12q24.33	AD	>20%	[154]
		*POLD1*	19q13.33			
	*AXIN2*-associated polyposis	*AXIN2*	17q24.1	AD	NA	[155]
**Hamartomatous**	Peutz–Jeghers syndrome (PJS)	*STK11*	19p13.3	AD	39%	[143]
	*PTEN* hamartoma tumor syndrome (PHTS)	*PTEN*	10q23.31	AD	11–20%	[156]
	Juvenile polyposis syndrome (JPS)	*BMPR1A*	10q23.2	AD	40–50%	[157]
		*SMAD4*	18q21.2		>50%	
**Serrated**	*RNF43*-associated serrated polyposis	*RNF43*	17q22	AD	NA	[158]
**Mixed**	*GREM1*-associated mixed polyposis	*GREM1*	15q13.3	AD	11–20%	[159]

AD: autosomal dominant; AR: autosomal recessive; NA: not assessed. * The percentages represent lifetime risks.

## Data Availability

Not applicable.
